# Prevalence and antifungal drug resistance of nosocomial *Candida* species isolated from two university hospitals in Egypt

**DOI:** 10.18502/cmm.7.1.6181

**Published:** 2021-03

**Authors:** Amira M. El-Ganiny, Nehal E. Yossef, Hend A. Kamel

**Affiliations:** 1 Microbiology and Immunology Department, Faculty of Pharmacy, Zagazig University, Zagazig, Egypt; 2 Microbiology Department, Faculty of Pharmacy and Pharmaceutical Industries, Sinai University, Kantara, Egypt

**Keywords:** Antifungal drug resistance, Azoles and caspofungin, *Candida albicans*, Non-*albicans*, Nosocomial infections

## Abstract

**Background and Purpose:**

There is a significant rise in morbidity and mortality of infections caused by *Candida*. *Candida* spp. infections are currently ranked fourth among nosocomial infections which are difficult to diagnose and refractory to therapy. Given the differences in susceptibility among various spp., identification of *Candida* spp. is an important step that leads to the selection of a suitable antifungal.

**Materials and Methods:**

A prevalence study was conducted on 122 *Candida* isolates. The *Candida* spp. were identified using Chromogenic agar and polymerase chain reaction (PCR). The antifungal susceptibility (AFS) of *Candida* spp. to amphotericin B, fluconazole, voriconazole, and caspofungin was determined by the disc diffusion method.

**Results:**

In total, 122 *Candida* clinical isolates were investigated in this study. *Candida albicans* with 57.4% (70 isolates) had the highest prevalence rate, while 52 isolates (42.6%) were non-*albicans* *Candida* species (NAC). The NAC include *Candida krusei* (20.4%), *Candida tropicalis* (6.5%), *Candida parapsilolsis* (5.7%), *Candida dubliniensis* (4.9%), and *Candida glabrata* (4.9%). The AFS showed that the resistance rates of *Candida* spp. to fluconazole and voriconazole were 13.1% (16 isolates) and 9.8% (12 isolates), respectively. Moreover, only five isolates (4.1%) were resistant to caspofungin. Furthermore, there was no resistance against amphotericin B. The spp. that showed the highest resistance were *C. glabrata* and *C. tropicalis*, while the lowest resistance was observed in *C. albicans* and *C. dubliniensis*.

**Conclusion:**

In conclusion, rapid identification of clinical *Candida* isolates and standard AFS are essential procedures for controlling the rise of resistant NAC spp. in clinical settings. Usage of fluconazole should be restricted, especially in patients with recurrent *Candida* infections.

## Introduction

There has been a significant rise in morbidity and mortality of infections caused by *Candida*, especially in recent years, due to the continuous rise in the number of infections, particularly in hospitalized patients [ 1 ]. Hospital-acquired (nosocomial) infection is more prevalent in certain patient groups, including transplant recipients, cancer patients, and other patients who receive immunosuppressive therapy [ 2 ]. Nosocomial infection is defined as an infection that is not apparent when the patient is admitted to the hospital but appears at least 48 h after the admission [ 3 ]. 

*Candida* spp. cause infections that range from non-life-threatening mucosal illnesses to invasive fatal infections, such as bloodstream infections [ 4 ]. Nosocomial infection by *Candida* is a problematic issue worldwide. *Candida* spp. are currently ranked fourth and sixth among the causative agents of nosocomial bloodstream infections (BSI) in the USA and Europe, respectively [ 2 , 5 ]. Egypt showed the highest *Candida* BSI burden, compared to the other Middle East neighboring countries [ 6 ]. 

*Candida* identification procedures usually start with the germ tube test that can differentiate *C. albicans* and *C. dubliniensis* from other *Candida* spp. [ 7 ]. Further tests, such as culturing on cornmeal agar, carbohydrate fermentation, and carbohydrate assimilation tests, are performed for the detection of other spp. [ 8 ]. Moreover, several chromogenic culture media have been developed to allow rapid identification of mixed *Candida* spp. [ 9 ]. The molecular approaches have the potential to detect *Candida* spp. with increased sensitivity and specificity [ 10 ]. The sequences of internal transcribed spacer (ITS) regions 1 and ITS2 have been used in various Polymerase chain reaction (PCR)-based methods for identification of medically important *Candida* spp. [ 11 ].

Identification of *Candida* to the spp. level is an important step that leads to the selection of suitable antifungals. Changes in *Candida* spp. distribution may impact treatment recommendations due to differences in susceptibility to antifungal agents among different spp. [ 12 ]. Antifungal agents available for the treatment of invasive candidiasis are restricted to polyenes, azoles, and the most recent echinocandin class [ 13 ]. Emergence of multidrug-resistant strains that are insensitive to several classes of antifungals is a major concern worldwide [ 14 ]. Studies on the prevalence rate of infections and antifungal susceptibility testing can help the selection of a proper treatment strategy that limits the emergence of resistance [ 15 ].

In the current study, different *Candida* spp. were identified in clinical specimens using Chromogenic agar and PCR-based methods. Furthermore, the antifungal drug resistance of the identified spp. was determined by the disc diffusion method.

## Materials and Methods

### Isolation and phenotypic identification of Candida species

All the clinical specimens were collected from different sources (blood, urine, and sputum) in clinical laboratories at Mansoura and Zagazig University Hospitals, Egypt. *Candida* identification started with a microscopical examination followed by growth on chromogenic agar and PCR [ 7 ]. The clinical specimens were collected under ethical standards and due to retrospective nature of the study, consent form were not applicable to the study. First, the specimens were inoculated on Sabouraud dextrose agar (SDA) plates and incubated at 37 °C for 24-72 h. The colonies were examined microscopically after Gram staining. Colonies that were proved microscopically to be *Candida*, were sub-cultured on HiChrome agar (HiMedia Laboratories, Mumbai, India) and incubated aerobically at 30 °C. They were inspected after 24-72 h, and the colony color was recorded and interpreted according to the manufacturer instructions. Briefly, *C. albicans* and *C. dubliniensis* gave pale green colonies, *C. krusei* gave purple fuzzy colony, *C. tropicalis* gave blue to purple colony, while other *Candida* spp. gave creamy white colonies.

### Confirmation of Candida species identification by Polymerase chain reaction

The DNA of *Candida* spp. was extracted by colony PCR method [ 16 ]. A pure colony of each isolate was picked up from SDA and inoculated into 30 uL of Tris-EDTA (TE) buffer. The mixture was heated at 100 °C in a water bath for 10 min and subsequently centrifuged at 10000 rpm for 2 min. Finally, the supernatants were transferred to a fresh Eppendorf tube. 

The primers used for the identification of *Candida* spp. were purchased from Operon Biotechnologies (GmbH Biocompus Cologne, Germany). Table 1 summarizes the sequences of these primers. During the PCR, the species-specific primers formed a pair with the universal primer UNI2 for all tested *Candida* spp., with the exception of *C. lusitaniae*, in which species-specific primer (Clus) paired with UNI1 [ 11 ].

**Table1:** Universal and species-specific primers used in the identification of *Candida* spp. and size of amplified fragments

*Candida* spp.	Primer	Sequence (5' to 3')	Size (bp)
Universal primers	UNI1	GTCAAACTTGGTCATTTA	
	UNI2	TTCTTTTCCTCCGCTTATTG	
*C. albicans*	Calb	AGCTGCCGCCAGAGGTCTAA	446
*C. krusei*	Ckru	CTGGCCGAGCGAACTAGACT	169
*C. tropicalis*	Ctro	GATTTGCTTAATTGCCCCAC	507
*C. parapsilolsis*	Cpar	GTCAACCGATTATTTAATAG	370
*C. dubliniensis*	Cdub	CTCAAACCCCTAGGGTTTGG	217
*C. glabrata*	Cgla	TTGTCTGAGCTCGGAGAGAG	839
*C. guilliermondii*	Cgui	TTGGCCTAGAGATAGGTTGG	512
*C. lusitaniae*	Clus	TTCGGAGCAACGCCTAACCG	329

The target DNA was amplified in a 20 μL reaction mixture containing 1 μL DNA samples, 10 μL of my Taq red mix (Bioline Co., UK), 1 μL of each forward and reverse primers, and up to 20 μL of nuclease-free water. The cycling conditions included heating at 95 °C for 3 min, followed by 30 cycles at 94 °C for 60 sec, 52 ºC for 30 sec, 65 °C for 45 sec, and finally heating at 65 °C for 7 min [ 7 ]. The PCR products, as well as 100 bp molecular DNA ladder (Bioline Co., UK), were separated on 1% agarose gel, stained with ethidium bromide (Merck, Hohenburnn, Germany) and visualized by a UV transilluminator. 

### Determination of antifungal susceptibility by disk diffusion method

*Candida* isolates were tested for their susceptibility to different antifungal agents by disk diffusion method according to Clinical and Laboratory Standard Institute guidelines [ 17 ]. Briefly, Three to five well-isolated colonies were inoculated into 4-5 mL Sabouraud dextrose broth (Oxoid, Hampshire, England) and the broth was incubated for 24 h at 37°C. The turbidity of the suspension was adjusted to the turbidity of 0.5 McFarland turbidity standards (10^7^ cells/mL).

A sterile cotton swab was dipped into the prepared suspension (within 15 min of adjusting the turbidity) and rotated firmly against the inside of the tube to remove excess fluid. Afterward, it was used to streak over Muller Hinton agar (MHA) (Oxoid, Hampshire, England) plate (containing 2% glucose and 0.5 µg/mL methylene blue). The antifungal disks were placed on the MHA plates using sterile forceps. Disks were pressed firmly against the agar surface to ensure contact and antifungal diffusion. The plates were inverted and incubated at 37 °C for 24-48 h. The diameter of inhibition zones around each antifungal disk was measured in millimeters and interpreted as susceptible, intermediate, or resistant according to interpretative criteria of CLSI [ 17 ]. 

The tested disks included amphotericin B (AMB, 10μg) as polyene drug, fluconazole (FLU, 25μg) as representative of the first generation azoles, voriconazole (VOR, 1μg) as representative of the second generation azoles, and caspofungin (CASP, 5μg) as representative of the echinocandin drug class. The antifungal disks were obtained from Bioanalyse, (Ankara, Turkey), and the standard strain, *C. albicans* ATCC 10231, was used as the reference strain.

## Results

### Identification of Candida species isolates

**Figure 1:**
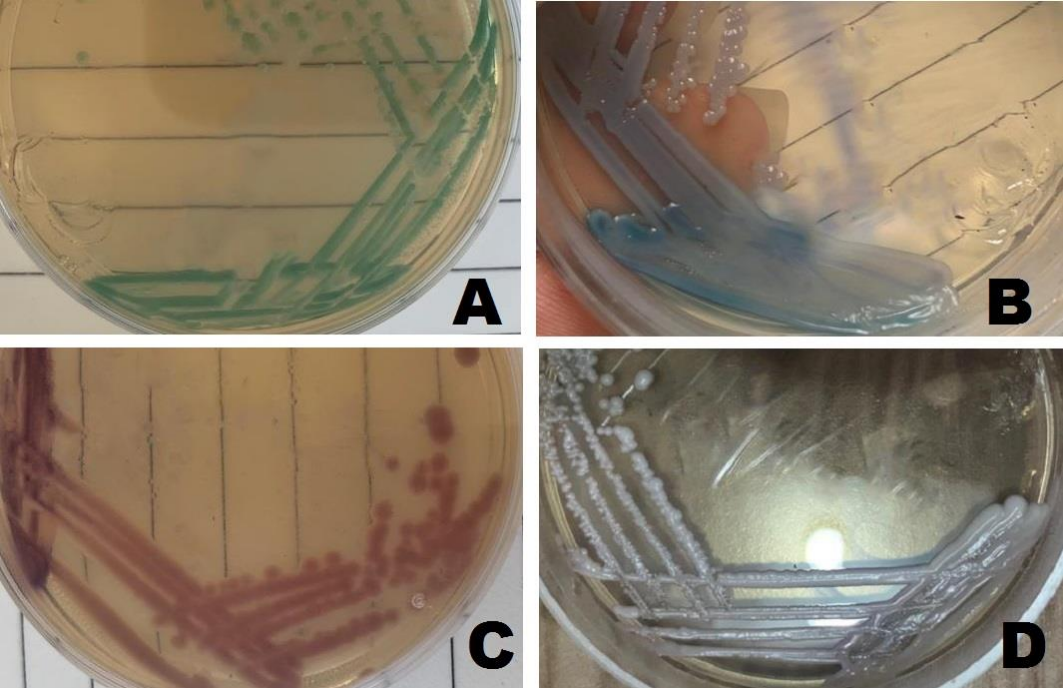
Identification of *Candida* spp. using Chromogenic agar. A: *C. albicans* and *C. dubliniensis*, B: *C. tropicalis*, C: *C. krusei*, D: other *Candida* spp

In total, 122 non-duplicate *Candida* clinical isolates were identified in the present study. These isolates were from different clinical sources; 21 (17.2 %), 45 (36.9%), 56 (45.9 %) isolates were from blood, urinary tract infection, and respiratory tract infection, respectively. In HiChrome agar, 72 isolates (59%) produced light green colonies (*C. albicans* or *C. dubliniensis*), 22 isolates (18%) were identified as *C. krusei* (gave purple colonies), eight (6.5%) were identified as *C. tropicalis* (gave blue colonies), and 20 (16.3%) produced white colonies and were identified as other *Candida* spp. (Figure 1)

**Figure 2:**
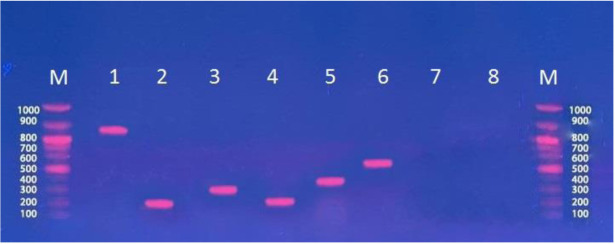
Polymerase chain reaction products for identification of *Candida* spp. Lane M had molecular weight marker, lane (1): *C. albicans* gave a single band of 446 bp, lane (2): *C. krusei* gave a single band of 169 bp, lane (3): *C. tropicalis* gave a single band of 507 bp, lane (4): *C. parapsilosis* gave a single band of 370 bp, lane (5): *C. dubliniensis* gave a single band of 217 bp, lane (6): *C. glabrata* gave a single band of 839 bp, lanes (7 and 8): negative results for *C. guilliermondi* or *C. lusitaniae*.

According to the PCR results that are presented in Figure 2, 70 isolates (57.4%) gave a single band of 446 bp and were identified as *C. albicans*, while 52 isolates (42.6%) were non-*albicans*. The non-*albicans* *Candida* spp. included 20.4% *C. krusei* (25 strains gave a single band of 169 bp), 6.5% *C. tropicalis* (eight isolates gave a single band of 507 bp), 5.7% *C. parapsilosis* (seven isolates gave a single band of 370 bp), 4.9% *C. dubliniensis* (six isolates gave a single band of 217 bp), and 4.9% *C. glabrata* (six isolates gave a single band of 839 bp). In the present study, none of the isolates were *C. guilliermondi* or *C. lusitaniae*.

### Susceptibility to antifungals

**Figure 3:**
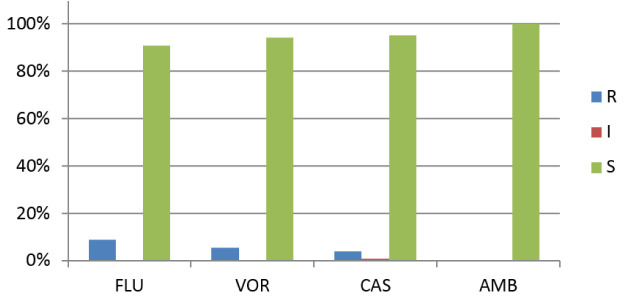
Antifungal susceptibility testing of *Candida* isolates. FLU: fluconazole, VOR: voriconazole, CAS: caspofungin, AMB: Amphotericin B, R: resistant, I: intermediate resistance, S: sensitive isolates.

The antifungal susceptibility testing of the 122 *Candida* strains revealed resistance to three antifungal drugs (FLU, VOR, and caspofungin). The data presented in Figure 3 showed that the percentages of resistance to FLU and VOR were 13.1% (16 isolates) and 9.8% (12 isolates), respectively. For caspofungin, five isolates (4.1%) were resistant, and only one isolate (0.8 %) showed intermediate resistance. It should be mentioned that there was no resistance to amphotericin B and *C. albicans* ATCC 10231 was sensitive to all tested antifungals.

In total, 22 isolates were resistant to antifungals, and each strain showed resistance to only one drug class (Table 2). Based on the findings, seven isolates of *C. albicans* showed resistance to azoles with no resistance to caspofungin, five *C. krusei* isolates were resistant (four were resistant to azoles and one to caspofungin), four *C. tropicalis* were resistant (one was resistant to azole and three to caspofungin), four *C. glabrata* were resistant to azoles only. Furthermore, one *C. parapsilolsis* strain was resistant to caspofungin and one *C. dubliniensis* strain was intermediately resistant to caspofungin. 

**Table2:** Number and percentage of *Candida* isolates that showed resistance to azoles and caspofungin

*Candida* spp.	Total number of isolates	Number of resistant isolates (%)	FLU	VOR	CAS
*C. albicans*	70	7 (10%)	7 (10%)	6 (8.6%)	-
*C. krusei*	25	5 (20%)	4 (16%)	3 (12%)	1 (4%)
*C. tropicalis*	8	4 (50%)	1 (12.5%)	1(12.5%)	3 (37.5%)
*C. parapsilolsis*	7	1 (14.3%)	-	-	1 (14.3%)
*C. dubliniensis* *	6	1 (16.7%)	-	-	1(16.7%)*
*C. glabrata*	6	4 (66.7%)	4(66.7%)	2 (33.3%)	-
Total	122	22 (18%)	16 (13.1%)	12 (9.8%)	5 (4.1%)

FLU, fluconazole, VOR: voriconazole, CAS: caspofungin

* Intermediate resistance

## Discussion

The incidence of fungal infections with high morbidity and mortality has increased globally due to the limited antifungal arsenal and the high toxicity of some drugs. Only five antifungal drug classes are available, including polyenes, azoles, and allylamines that target ergosterol in the cell membrane, pyrimidine analogs that target DNA synthesis, and the new echinocandin class that targets β-glucan in the fungal cell wall [ 18 ]. *Candida* spp. are currently ranked fourth among nosocomial BSIs in the USA, accounting for 8-10% of all BSIs acquired in hospitals [ 19 ]. *Candida* infections are ranked as the sixth most common cause of nosocomial infection in Europe [ 2 , 5 ]. Furthermore, *Candida* BSIs are more prevalent in Egypt than in other Middle East countries [ 6 ]. 

Given the drastic increase in non-*albicans* *Candida* species (NAC) infections and the distinct antifungal susceptibility pattern of these spp., accurate identification becomes essential for proper clinical management [ 19 ]. The current study aimed to identify and determine the antifungal susceptibility of different *Candida* spp. from clinical infections.

Both Chromogenic agar and PCR were used for the identification of *Candida* to the spp. level in this study. Chromogenic agar is an economic and simple method, and in this study, the sensitivity of chromogenic media in the identification of *Candida* spp. was about 95% which was consistent with the previously reported 96.3% and 97.5% in a study conducted in Egypt [ 7 , 20 ]. However, Chromogenic agar was unable to differentiate *C. albicans* from *C. dubliniensis* and some spp. have no distinct color on it.

Obviously, the PCR method showed better sensitivity in the detection of *Candida* spp., which was in agreement with the previously reported results [ 7 , 11 ]. In the present study, the use of species-specific primers allowed the differentiation of several *Candida* spp. This method makes it possible to identify the spp. that have non-morphologic, cultural, and biochemical characteristics. 

According to the results of the current study, *C. albicans* was the most prevalent spp. (57.4%) which was comparable to the 62.9 % reported in vaginal infections [ 21 ]. Kadry et al. [ 7 ] reported a prevalence rate of 70% which is slightly higher than the results of the present study. Moreover, according to a study performed in Egypt, the prevalence rate of *C. albicans* in BSIs was 40%, which is lower than the findings of the present study [ 22 ]. These differences in prevalence could be attributed to the variety of sources from which the clinical samples were isolated.

The NAC levels in this study (42.6%) are in line with the values in the literature that denote the epidemiological shift of *Candida* pathogens in the last few decades [ 2 ]. Nevertheless, this shift appears to be rising with time as the recently reported prevalence of NAC is around or even exceeds 50% in some cases [ 6 , 22 , 23 ]. The extensive use of antifungals for prophylaxis has become the leading cause of colonization of NAC and increase of resistance to antifungal drugs [ 24 ].

It was reported previously that *C. tropicalis* and *C. parapsilosis* were the most prevalent *Candida* spp. after *C. albicans* [ 24 ]. However, in this study, *C. krusei* ranked second after *C. albicans* with a prevalence rate of about 20%, followed by *C. tropicalis* (6.5%) and *C. parapsilosis* (5.7%). Moreover, a similar prevalence rate for *C. krusei* was recently reported in Egypt [ 6 , 22 ]. A lower prevalence of *C. krusei* was reported (10.7%) in vaginal infections [ 21 ], while it had a prevalence rate of 46% in leprosy patients [ 25 ]. Besides, it was reported that *C. krusei* was highly associated with FLU exposure [ 26 ]. Several studies have also reported variable prevalence rates for *C. tropicalis* (8-40%) and *C. parapsilosis* (3-14%) in different clinical sources [ 27 ].

In the present study, both *C. dubliniensis* and *C. glabrata* had a prevalence rate of 4.9%. Sharma et al. reported a prevalence rate of 0.5-6.3% for *C. glabrata* [ 28 ], while Yang et al. detected a rate of 13-20% in different clinical samples [ 29 ]. Besides, various prevalence rates of *C. dubliniensis* (0-5.5%) were observed in different clinical sources [ 29 ] and different countries around the world. Accordingly, 1.4% was reported in Egypt [ 30 ], 9% in Germany [ 31 ], and 11% in Sweden [ 32 ]. Overall, changes in spp. distribution are attributed to several factors, including different geographical regions, hospital-related factors, sources of the specimen, and type of antifungal therapy. 

Regarding antifungal susceptibility, in this study, the highest level of resistance was observed against FLU (13.1%) and VOR (9.8%). The spp. that showed FLU resistance were *C. glabrata* (66.6%), *C. krusei* (16%), *C. tropicalis* (12.5%), and *C. albicans* (10%). Furthermore, Khairat et al. [ 22 ] reported that azole resistance is higher in NAC spp., compared to *C. albicans* (44% versus 38.9%). The *C. glabrata* has intrinsically low susceptibility to azoles, and acquired azole resistance has been documented during treatment [ 33 ]. 

Moreover, *C. krusei* exhibits intrinsic resistance to FLU [ 34 ]. Reported FLU resistance in *C. tropicalis* is within the range of 0-83% and 4-9% in South Korea [ 35 ] and the USA [ 36 ], respectively. The FLU is known to be the most commonly prescribed antifungal [ 34 ], and its prolonged use in treating *Candida* infections has led to the emergence of resistance in all *Candida* spp. [ 12 ]. 

Caspofungin is the first echinocandin drug, and 60% of patients with candidemia are reported to have received an echinocandin drug. Resistance to echinocandins has evolved since 2005 but remains relatively low [ 37 ]. In this study, three spp. exhibited resistance to caspofungin, *C. tropicalis* (37.5%), *C. parapsilolsis* (14.3%), and *C. krusei* (4%). Only one *C. dubliniensis* strain was intermediately resistant to caspofungin, while *C. albicans* and *C. glabrata* did not show any resistance to caspofungin. Similarly, the results of previous studies have shown that *C. albicans* had no resistance against caspofungin [ 38 , 39 ]. The *C. dubliniensis* normally does not show elevated resistance to echinocandins [ 40 ], while *C. tropicalis* acquires resistance after short-term treatment with caspofungin [ 41 ]. Moreover, *C. parapsilosis* tends to be more tolerant to echinocandins [ 2 ].

The polyene drug AmB has been the most potent fungicidal drug for decades; however, its renal toxicity has limited its use, hence liposomal formulations of AmB are used to reduce its toxicity [ 42 ]. None of our isolates showed resistance to AmB, which is in line with the results of previous studies performed in Egypt which reported either no resistance [ 39 ] or very low (3%) resistance to AmB [ 22 ]. It is well known that *Candida* rapidly develops resistance to azoles and echinocandins. Nevertheless, resistance to AmB remains extremely rare despite decades of use [ 43 ].

## Conclusion

*Candida* spp. are responsible for many fungal infections in humans and a noticeable increase of NAC infections. Changes in *Candida* spp. distribution may impact treatment recommendations due to differences in susceptibility to antifungals among the spp. Regarding azoles and echinocandin, intrinsic resistance in some spp. and acquired resistance in other spp. were observed. The AmB remains the gold standard drug for the treatment of *Candida* infections as resistance to it is very rare. Finally, accurate identification of spp. and standard antifungal susceptibility testing are essential procedures for controlling the rise of resistant *Candida* strains.

## Authors’ contribution

A.M.E. conceived the idea of this research, participated in the data analysis and revised the manuscript. H.A.K. performed the experiments, participated in data analysis and wrote the initial manuscript draft. N.Y. participated in the initial idea of this research, provided intellectual input and edited the manuscript. All authors read and approved the final manuscript. 

## Financial disclosure

The authors declare no financial disclosure.
